# DDIT4 mediates the proliferation-promotive effect of IL-34 in human monocytic leukemia cells

**DOI:** 10.1097/BS9.0000000000000069

**Published:** 2021-04-27

**Authors:** Xiaoqian Lv, Yuting Hu, Lina Wang, Dongyue Zhang, Hao Wang, Yibo Dai, Xiaoxi Cui, Guoguang Zheng

**Affiliations:** State Key Laboratory of Experimental Hematology, National Clinical Research Center for Blood Diseases, Institute of Hematology & Blood Diseases Hospital, Chinese Academy of Medical Sciences & Peking Union Medical College, 288 Nanjing Road, Tianjin 300020, China

**Keywords:** IL-34, DDIT4, Monocytic leukemia, Proliferation

## Abstract

*Interleukin 34 (IL-34) is a cytokine that shares the receptor with colony-stimulating factor 1 (CSF-1).* IL-34 is involved in a broad range of *pathologic processes including* cancer. *We previously demonstrated* that IL-34 promoted the proliferation and colony formation of human acute monocytic leukemia (AMoL) cells. However, the mechanism has not been elucidated. Here, by analyzing the gene profiles of Molm13 and THP1 cells overexpressing IL-34 *(Molm13-IL-34 and THP1-IL-34)*, upregulation of the DNA damage-inducible transcript 4 (DDIT4) was detected in both series. Knockdown of DDIT4 *effectively inhibited the proliferation, promoted apoptosis and colony formation in Molm13-IL-34 and THP1-IL-34* cells. Our results suggest that DDIT4 mediates the proliferation-promotive effect of IL-34 whereas does not mediate the promotive effect of IL-34 on colony formation in AMoL cells.

## 1 INTRODUCTION

Leukemia is the hematopoietic malignancy characterized by clonal expansion of immature blood cells. Sophisticated mechanisms, which are mediated by both internal and microenvironmental factors, are involved in the genesis and progression of leukemia.^1–3^ Colony-stimulating factor 1 (CSF-1), also known as the macrophage CSF (M-CSF), is highly expressed in leukemia, especially acute myeloid leukemia (AML).^4^ CSF-1 not only directly stimulates leukemia cells, such as acute monocytic leukemia (AMoL) cells but also recruits and activates leukemia associated-macrophages.^5,6^*IL-34 is a cytokine* that shares the same receptor with CSF-1, CSF-1R, and the CSF-1/*IL-34-*CSF-1R axis was suggested.^7^ Under physiologic conditions, IL-34 promotes the survival, proliferation, and differentiation of monocytes,^4,8^ maintains the function of macrophages and participates in the developmental process of various tissues.^9–12^ IL-34 is also involved in many pathologic processes ranging from inflammation and autoimmunity to cancer.^13^ Nevertheless, the effect of IL-34 in leukemia cells has not been well established. We previously demonstrated that IL-34 promoted the malignant biological behavior of human AMoL cells.^14^ However, the mechanism has not been elucidated.

DNA damage-inducible transcript 4 (DDIT4) is activated under various cellular stresses including DNA damage, hypoxia, oxidative stress, and starvation.^15^ DDIT4 inhibits the activity of the mammalian target of rapamycin (mTOR) and participates in the regulation of diverse cell functions including proliferation, apoptosis, and differentiation.^16–18^ DDIT4 has been implicated in several human solid tumors. However, its effect seems to be cell type-dependent.^19–21^ In AML, DDIT4^high^ cases have a worse prognosis than DDIT4^low^ cases^22^ although bloodspot shows that AML cases express a lower level of DDIT4 than normal donors (http://servers.binf.ku.dk/bloodspot/). Hence, complicated mechanisms should be involved. It has been documented that DDIT4 mediates the effect of different cytokines. IL-10 induces DDIT4 expression to suppress mTOR activity^23^ whereas IL-6 reduces DDIT4 expression and further results in IL-6-induced activation of mTOR signaling.^24^ It is worth noting that mTOR/S6 kinase is also downstream of CSF-1R upon CSF-1 activation.^25^ However, whether DDIT4 mediates the effect of IL-34 in AMoL has not been documented.

In this study, the gene profile of AMoL cells overexpressing IL-34, that is, *Molm13-IL-34 and THP1-IL-34* cells, was first analyzed. DDIT4 was upregulated in both series. Then, the effect of DDIT4 in *Molm13-IL-34 and THP1-IL-34* cells was investigated by silencing DDIT4. Knockdown of DDIT4 *effectively inhibited the proliferation whereas promoted apoptosis and colony formation in both* cells.

## 2 RESULTS

### 2.1 Gene expression profile of Molm13-IL-34 and THP1-IL-34 cells

**Figure 1:**
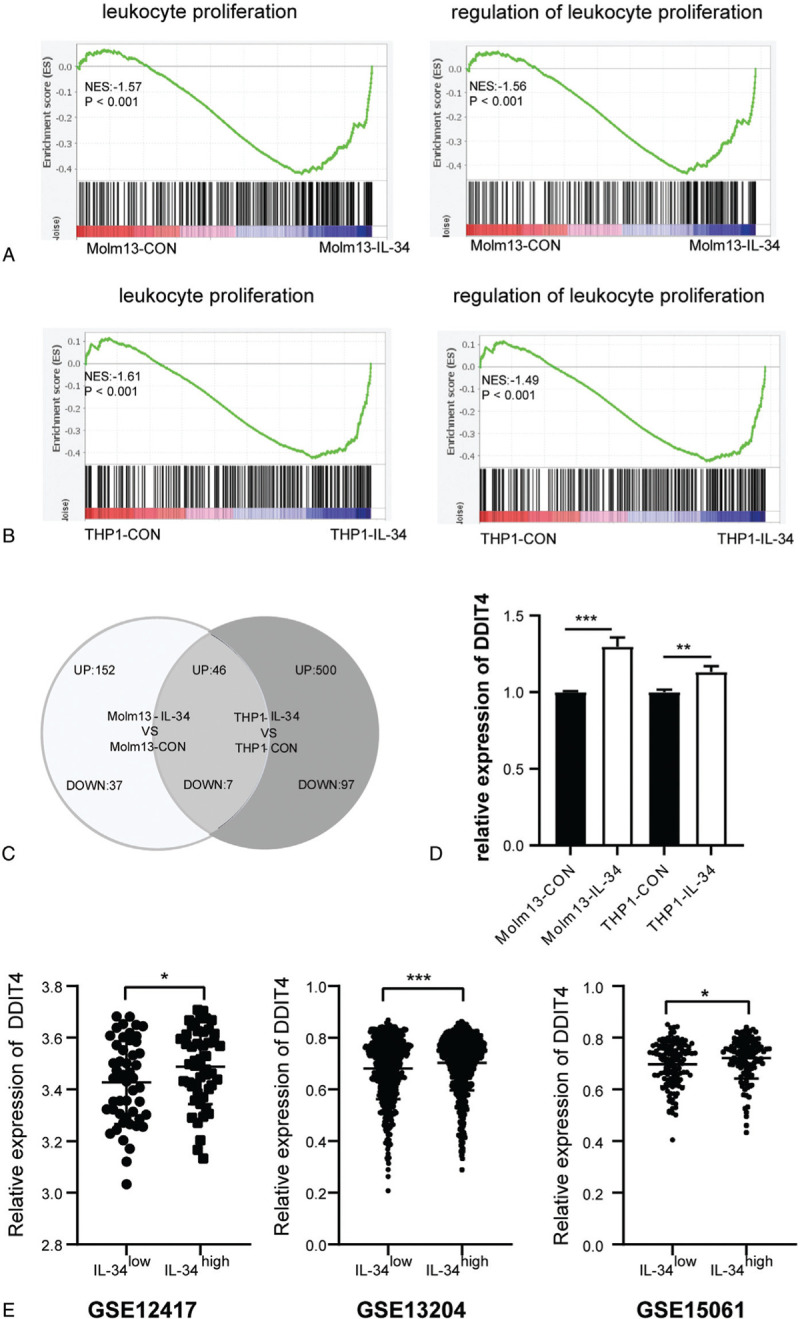
Screening key molecules mediating the effect of IL-34 in Molm13-IL-34 and THP1-IL-34 cells. Molm13-CON, Molm13-IL-34, THP1-CON, and THP1-IL-34 cells were collected and RNA-seq was performed. (A) and (B) All DEGs were analyzed by GSEA. Enriched annotations, leukocyte proliferation, and regulation of leukocyte proliferation are shown. (A) Molm13-IL-34 versus Molm13-CON. (B) THP1-IL-34 versus THP1-CON. (C) DEGs were further filtrated by FC ≥ 2.0 and FDR < 0.01. The Venn diagram shows the overlap of these DEGs. (D) The relative expression of DDIT4 was analyzed by RT-PCR. (E) Correlation between the expressions of IL-34 and DDIT4 was studied from the GSE12417, GSE13204, and GSE15061 datasets. ^∗^*P* < 0.05; ^∗∗^*P* < 0.01; and ^∗∗∗^*P* < 0.001. DDIT4 = DNA damage-inducible transcript 4; DEGs = differentially expressed genes, FC = fold change, FDR = false discovery rate GSEA = gene set enrichment analysis, RT-PCR = reverse transcription-polymerase chain reaction.

We previously demonstrated that overexpression of IL-34 in human AMoL cells increased cell proliferation and colony-forming while promoted cell differentiation into the monocyte-macrophage lineage.^14^ To explore the mechanism, Molm13-CON, Molm13-IL-34, THP1-CON, and THP1-IL-34 cells were collected for RNA-seq analysis. All differentially expressed genes (DEGs) were undergone gene set enrichment analysis (GSEA) and the annotations of leukocyte proliferation and regulation of leukocyte proliferation were enriched in both Molm13-IL-34 and THP1-IL-34 cells (Fig. 1A and B). Then, the filtrated DEGs (fold change [FC] ≥ 2.0, false discovery rate [FDR] < 0.01), that is, 152 up-regulated genes and 37 down-regulated genes in Molm13-IL-34 cells, 500 up-regulated genes, and 97 down-regulated genes in THP1-IL-34 cells, were further analyzed. Molm13-IL-34 and THP1-IL-34 cells shared 46 up-regulated genes and 7 down-regulated genes (Fig. 1C). The *DDIT4* gene, which is a negative regulator of mTOR, was 1 of the up-regulated genes. Reverse transcription-polymerase chain reaction (RT-PCR) experiments further verified that Molm13-IL-34 and THP1-IL-34 cells expressed higher levels of DDIT4 than the respective controls (Fig. 1D). Analysis of GSE12417, GSE13204, and GSE15061 datasets further confirmed the positive correlation between the expressions of IL-34 and DDIT4 (Fig. 1E). These results suggest that DDIT4 potentially mediates the effect of IL-34 in Molm13-IL-34 and THP1-IL-34 cells.

### 2.2 Knockdown of DDIT4 in Molm13-IL-34 and THP1-IL-34 cells

**Figure 2:**
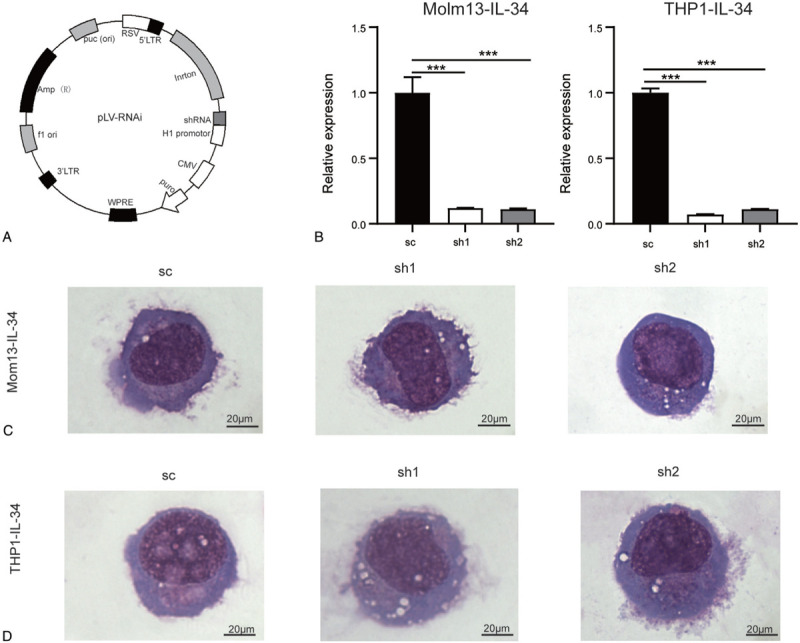
Knockdown of DDIT4 in Molm13-IL-34 and THP1-IL-34 cells. (A) The structure of recombinant vectors (pLV-DDIT4-sh1, pLV-DDIT4-sh2, and pLV-DDIT4-sc) is shown. (B) The relative expression of DDIT4 was determined by RT-PCR. (C)and (D) Cells were spun on slides and Wright staining was performed. The morphology of cells is shown. (C) Molm13-IL-34-sc, Molm13-IL-34-sh1, and Molm13-IL-34-sh2 cells. (D) THP1-IL-34-sc, THP1-IL-34-sh1, and THP1-IL-34-sh2 cells. Scale bar: 20 μm. Bars represent mean ± SD. ^∗∗∗^*P* < 0.001. DDIT4 = DNA damage-inducible transcript 4, SD = standard deviation.

To study whether DDIT4 mediates the effect of IL-34 in AMoL cells, the expression of DDIT4 was knocked down in Molm13-IL-34 and THP1-IL-34 cells. The shRNA or scramble fragment was cloned into the pLV-H1-EF1α-puro vector (Fig. 2A). The recombinant vectors were verified by DNA sequencing. Molm13-IL-34 and THP1-IL-34 cells were infected with these viruses. For short, the sc group represents the cells infected by pLV-DDIT4-sc while the sh1 and sh2 groups represent those infected by pLV-DDIT4-sh1 and pLV-DDIT4-sh2. The expression of DDIT4 was considerably lower in the sh1 or sh2 group than the sc group (Fig. 2B). No significant difference in cell size and morphology was detected among the three groups that originated from either Molm13-IL-34 cells (Fig. 2C) or THP1-IL-34 cells (Fig. 2D). These data suggest that DDIT4 is successfully knocked down in both Molm13-IL-34 and THP1-IL-34 cells. Besides, knockdown of DDIT4 has little effect on cell morphology.

### 2.3 Knockdown of DDIT4 in Molm13-IL-34 and THP1-IL-34 cells decreases proliferation and increases apoptosis

**Figure 3:**
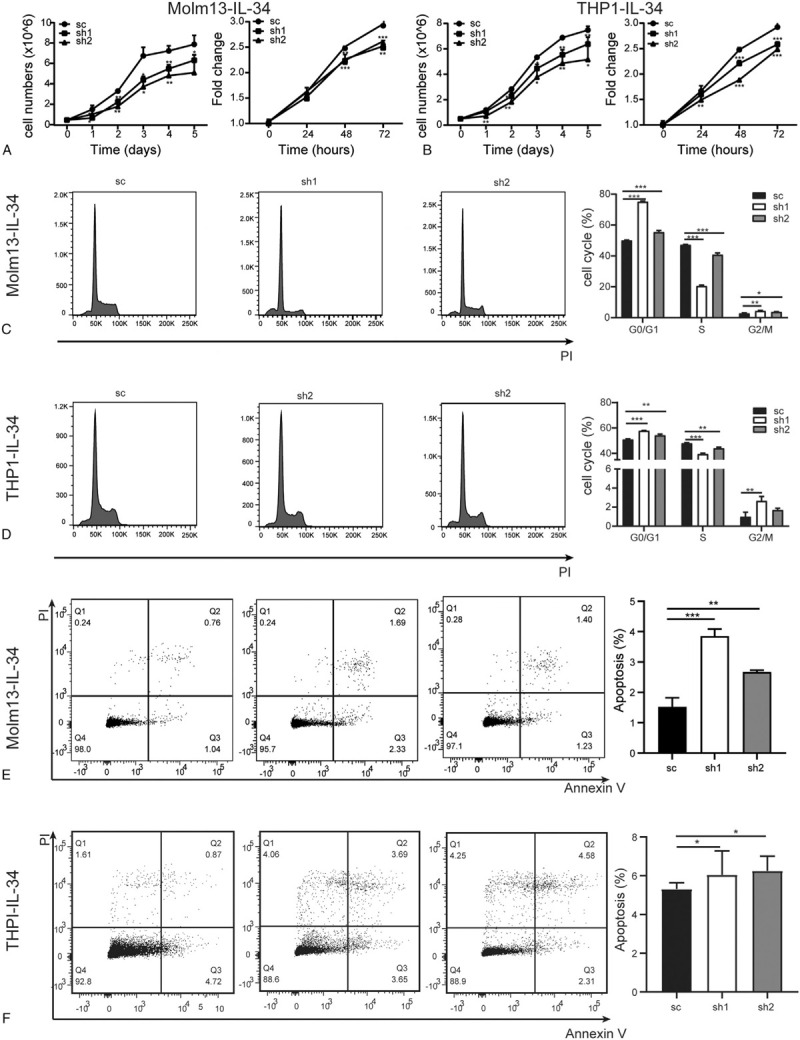
Knockdown of DDIT4 in Molm13-IL-34 and THP1-IL-34 cells decreases proliferation and increases apoptosis. (A) and (B) Cell proliferation was assessed by cell counting (left) and MTS (right) methods. (C) and (D) Cell cycle was assessed by PI-staining followed by flow cytometry analysis. The typical results are shown on the left and the percentage of G0/G1, S, and G2 phase cells are plotted on the right. (E) and (F) The apoptosis of cells was detected by Annexin V/PI staining. The typical results are shown on the left and the percentage of apoptotic cells is plotted on the right. (A), (C), and (E) Molm13-IL-34-sc, Molm13-IL-34-sh1, and Molm13-IL-34-sh2 cells. (B), (D), and (F) THP1-IL-34-sc, THP1-IL-34-sh1, and THP1-IL-34-sh2 cells. Bars represent mean ± SD. ^∗^*P* < 0.05; ^∗∗^*P* < 0.01; and ^∗∗∗^*P* < 0.001. DDIT4 = DNA damage-inducible transcript 4, MTS = 3-(4,5-dimethylthiazol-2-yl)-5-(3-carboxymethoxyphenyl)-2-(4-sulfophenyl)-2H-tetrazolium, SD = standard deviation.

Cell counting and 3-(4,5-dimethylthiazol-2-yl)-5-(3-carboxymethoxyphenyl)-2-(4-sulfophenyl)-2H-tetrazolium (MTS) experiments were used to determine the effect of DDIT4 on the proliferation of THP1-IL-34 and Molm13-IL-34 cells. The results demonstrated that knockdown of DDIT4 inhibited cell proliferation in Molm13-IL-34 (Fig. 3A) and THP1-IL-34 (Fig. 3B) cells. To further explore the effect of DDIT4 on cell cycle, propidium iodide (PI)-staining and flow cytometry analysis were performed. The proportion of G0/G1 phase cells was significantly increased while the proportion of S phase cells was distinctly decreased in the sh1 and sh2 groups (Fig. 3C and D).

The effect of DDIT4 on cell apoptosis in Molm13-IL-34 and THP1-IL-34 cells was evaluated by the Annexin V/PI staining. Knockdown of DDIT4 promoted cell apoptosis in Molm13-IL-34 and THP1-IL-34 cells (Fig. 3E and F).

The above data indicate that knockdown of DDIT4 in Molm13-IL-34 and THP1-IL-34 cells results in the decreased proliferation and increased apoptosis.

### 2.4 Knockdown of DDIT4 in Molm13-IL-34 and THP1-IL-34 cells increases colony formation

**Figure 4:**
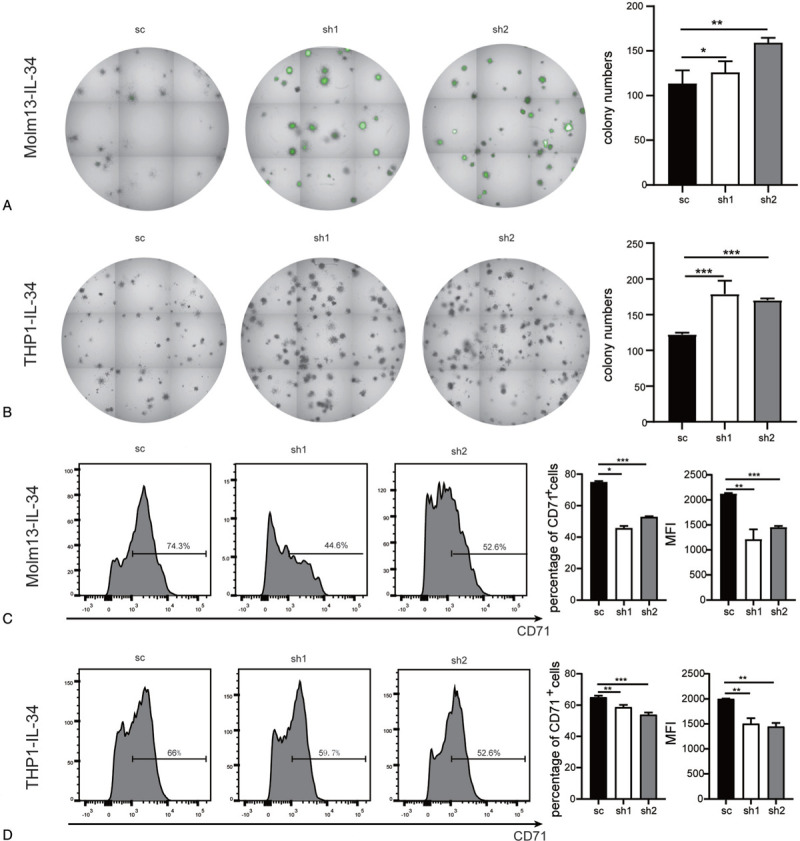
Knockdown of DDIT4 in Molm13-IL-34 and THP1-IL-34 cells increase colonies and decreases CD71 expression. (A) and (B) Colony-forming assay was performed and a high-content analysis system was used for colony analysis. The typical results are shown on the left and the number of colonies is plotted on the right. (C) and (D) The expression of CD71 was assessed by flow cytometry analysis. The typical results are shown on the left. The percentage of CD71^+^ cells and MFI are plotted on the right. (A) and (C) Molm13-IL-34-sc, Molm13-IL-34-sh1, and Molm13-IL-34-sh2 cells. (B) and (D) THP1-IL-34-sc, THP1-IL-34-sh1, and THP1-IL-34-sh2 cells. Bars represent mean ± SD. ^∗^*P* < 0.05; ^∗∗^*P* < 0.01; and ^∗∗∗^*P* < 0.001. DDIT4 = DNA damage-inducible transcript 4, SD = standard deviation.

The colony-forming potential of leukemia cells partly reflects the level of leukemia stem cells (LSCs). We next explored whether knockdown of DDIT4 affected the colony-forming potential of Molm13-IL-34 and THP1-IL-34 cells. The results showed that more colonies were detected in the sh1 or sh2 group than the sc group in both Molm13-IL-34 (Fig. 4A) and THP1-IL-34 (Fig. 4B) series. These results suggest that knockdown of DDIT4 in Molm13-IL-34 and THP1-IL-34 cells increase LSC level.

### 2.5 Knockdown of DDIT4 in Molm13-IL-34 and THP1-IL-34 cells decreases CD71 expression

To examine the effect of DDIT4 on the differentiation of Molm13-IL-34 and THP1-IL-34 cells, the expression of CD11b, CD14, and CD71 was detected by flow cytometry. CD11b is a differentiation marker of the myelo-monocytic lineage, CD14 is a binding site for lipopolysaccharide, and CD71 is a marker of immature cells. Compared with the sc group, the percentage of CD11b^+^ and CD14^+^ cells and the mean fluorescent intensity (MFI) showed little difference in the sh1 and sh2 groups of both Molm13-IL-34 and THP1-IL-34 series. A decrease in the percentage of CD71^+^ cells or MFI was detected in either sh1 or sh2 group of Molm13-IL-34 (Fig. 4C) and THP1-IL-34 (Fig. 4D) series.

## 3 DISCUSSION

IL-34 participates in both physiologic and pathologic processes. Evidence shows that IL-34 plays an important role in cancer through direct or indirect mechanisms.^26^ Tumor cell-derived IL-34 acts as an autocrine in CSF-1R-expressing tumor cells to provide a proliferative signal for tumor growth and survival signal for tumor cells against chemotherapy.^27^ IL-34 also acts as a paracrine to educate macrophages to tumor-associated macrophages, which in turn contribute to tumor progression.^28,29^ However, the role of IL-34 in leukemia and the related mechanism have not been well established. This work contributes to a better understanding of AMoL progression, elucidating the roles of IL-34 in leukemia and providing clues for potential targets against leukemia.

Our previous study explored the direct effect of IL-34 in human AMoL cells by overexpressing IL-34. IL-34 promotes the malignant biological behavior of AMoL cells by increasing cell proliferation and colony formation, and so on. To elucidate the mechanism, RNA-seq was performed. *DDIT4* was upregulated in Molm13-IL-34 and THP1-IL-34 cells, which were verified by RT-PCR. To further confirm the correlation, GSE datasets were analyzed. The IL-34^high^ group expressed a higher level of DDIT4 than the IL-34^low^ group. These results suggest the positive correlation between the expressions of IL-34 and DDIT4, which implies that DDIT4 may mediate the effect of IL-34 in AMoL cells. Hence, we studied how DDIT4 mediated the effect of IL-34.

DDIT4 is regulated by multiple signal pathways to exert different effects under physiologic and pathologic conditions. The effect of DDIT4 on malignancies has been documented. DDIT4 promotes the progression of ovarian cancer, gastric cancer, and pancreatic cancer, and so on.^30–32^ However, DDIT4 inhibits cell proliferation through the inactivation of mTOR signaling and reduction of HIF-1α instability in triple-negative breast cancer and HER2 phenotypes.^20^ In leukemia, although the expression of DDIT4 is lower in AML than normal donors, DDIT4^high^ patients have a worse prognosis than DDIT4^low^ cases.^33^ Here, we explored whether DDIT4 mediated the effect of IL-34 by knocking down DDIT4 in AMoL cells overexpressing IL-34. Knockdown of DDIT4 suppresses *the proliferation whereas promotes the apoptosis and colony formation in Molm13-IL-34 and THP1-IL-34 cells*.

Proliferation and apoptosis are two major characteristics of malignant cells.^34^ Overexpression of IL-34 in AMoL *cells promotes cell proliferation.* Proliferation-related annotations are enriched in RNA-seq and GSEA analyses. Furthermore, knockdown of DDIT4 in *both* Molm13-IL-34 and THP1-IL-34 cells attenuates *the proliferation-promotive effect and promotes cell apoptosis.* DDIT4 has been implicated in cell proliferation. Overexpression of DDIT4 promotes cell proliferation in human ovarian epithelial cells.^31^ Reduced DDIT4 level can sensitize cells to apoptosis, whereas the elevated level of DDIT4 induced by hypoxia or overexpression desensitizes cells to apoptotic stimulation.^21^*These results suggest that* DDIT4 mediates the proliferation-promotive effect of IL-34 in AMoL cells.

The malignant cells are heterogeneous and colony-forming potential is important *in vitro* marker for the malignant phenotype since it partly reflects the level of tumor stem cells or LSCs in the population.^35^ Malignant cells with high colony formation potential are commonly considered to be more malignant. Overexpression of IL-34 increases the number and size of colonies in AMoL cells.^14^ However, knockdown of DDIT4 does not suppress but further promotes colony formation in AMoL cells. This result indicates that knockdown of DDIT4 cannot attenuate the effect of IL-34 on colony formation in AMoL cells. Therefore, DDIT4 does not mediate the promotive effect of IL-34 on colony formation in AMoL cells. Further work should be done to unravel the molecular mechanism of how IL-34 affects colony formation in AMoL cells.

In summary, knockdown of DDIT4 effectively inhibits the proliferation, promotes apoptosis and colony formation in AMoL cells. DDIT4 mediates the proliferation-promotive effect of IL-34 whereas does not mediate the promotive effect of IL-34 on the colony formation in AMoL cells.

## 4 MATERIAL AND METHODS

### 4.1 Vectors and reagents

The lentivirus vector PLV-H1-EF1α-period was derived from Biosettia (USA). Fetal bovine serum (FBS), trypsin, penicillin/streptomycin, OPTI-MEM, sodium pyruvate, non-essential amino acids (NEAA), and l-glutamine were purchased from Gibco (USA). The SYBR Green PCR kit was acquired from TaKaRa Biotech (China). The EcoR I and Xba I restriction endonucleases were obtained from New England BioLabs (UK). DMEM and RPMI 1640 were purchased from Neuronbc (China). The H4434 was acquired from Stem Cell Technologies (Canada). Annexing-V/PI kit and the antibodies against human CD11b (PE), CD14 (APC), and CD71 (PE) were purchased from BioLegend (USA).

### 4.2 Construction of recombinant plasmids

DDIT4-sh1 targeting DDIT4, and the control shRNA, DDIT4-sc were designed using RNAi designer at Biosettia's website (http://biosettia.com/support/shrna-designer). DDIT4-sh2 targeting DDIT4 was designed using RNAi designer at Sigma-Aldrich's website (https://www.sigmaaldrich.com/life-science/functional-genomics-and-rnai/shrna/individual-genes.html). The sequences of them were 5′-AAAAGCTTCCGAGT CATCAAGAATTGGATCCAATTCTTGATGACTCGGAAGC-3′ (DDIT4-sh1), 5′-AAAA TGATGCCTAGCCAGTTGGTAATTGGATCCAATTACCAACTGGCTAGGCATCA-3′ (DDIT4-sh2), and 5′-AAAAGCAGTTATCTGGAAGATCAGGTTGGATCCAACCTGATC TTCCAGATAACTGC-3′ (DDIT4-sc), respectively. The single-strand DNA oligo was annealed to form a two-strand oligo before ligated to the linearized vector PLV-H1-EF1α-puro to construct shRNA vectors. The recombinant lentiviruses were named pLV-DDIT4-sh1, pLV-DDIT4-sh2, and pLV-DDIT4-sc, respectively.

### 4.3 Cell lines and cell culture

The establishment of Molm13 and THP1 cells overexpressing IL-34, Molm13-IL-34, and THP1-IL-34, and the respective control cell lines, Molm13-CON and THP1-CON was described previously.^14^ To investigate the effect of DDIT4, THP1-IL-34, and Molm13-IL-34 cells were infected with pLV-DDIT4-sc, pLV-DDIT4-sh1, or pLV-DDIT4-sh2. After screening, puromycin-resistant cells were used for further assay and named THP1-IL-34-sc, THP1-IL-34-sh1, THP1-IL-34-sh2, Molm13-IL-34-sc, Molm13-IL-34-sh1, and Molm13-IL-34-sh2, respectively.

The above cells were maintained in RPMI 1640 medium supplemented with 10% FBS, penicillin (100 U/ml), and streptomycin (100 μg/ml). HEK293T cells were cultured in DMEM medium supplemented with 10% FBS, sodium pyruvate, NEAA, and l-glutamine. All cells were cultured in a humidified incubator at 37°C and 5% CO_2_ atmosphere.

### 4.4 GSE datasets and data analysis

The GSE datasets were downloaded and used to study the correlation between the expressions of IL-34 and DDIT4. The GSE12417 dataset (https://www.ncbi.nlm.nih.gov/geo/query/acc.cgi?acc=GSE12417) includes 242 samples from AML patients with cytogenetically normal karyotype. The GSE13204 dataset (https://www.ncbi.nlm.nih.gov/geo/query/acc.cgi?acc=GSE13204) contains samples from 2023 patients with blood diseases and 73 healthy donors. The GSE15061 dataset (https://www.ncbi.nlm.nih.gov/geo/query/acc.cgi?acc=GSE15061) comprises samples from 202 AML, 164 myelodysplastic syndrome and 69 healthy donors. For each dataset, based on the expression level of IL-34, the IL-34^high^, and IL-34^low^ groups contained the top 30% and bottom 30% samples, respectively.

### 4.5 RNA sequencing (RNA-seq) and data analysis

Molm13-CON, Molm13-IL-34, THP1-CON, and THP1-IL-34 cells were sorted by flow cytometry. RNA-seq was carried out in the Beijing Genomics Institute (BGI) following standard protocols on BGISEQ-50. RNA-seq data of leukemia cell lines are available in the National Center for Biotechnology Information Gene Expression Omnibus database under the accession number GSE163969. All DEGs were analyzed by GSEA. For further analysis, DEGs were filtrated by FC ≥ 2.0 and FDR < 0.01.

### 4.6 cDNA synthesis and real-time PCR

Cells were harvested and total RNA was extracted using Trizol Reagent (Invitrogen, USA). Then, total RNA was reversely transcribed using *EasyScript*^®^ One-Step gDNA Removal and cDNA Synthesis SuperMix (TransGen Biotech, China) following the manufacturer's protocols. Real-time PCR was performed using SYBR Green Kit and QuantStudio 5 (Thermo Fisher Scientific, USA). The expression level of target genes was obtained from at least 3 independent experiments by calculating the RQ value using the ^ΔΔ^Ct method [^ΔΔ^Ct = (Ct_TARGET_ − Ct_GAPDH_)_sample_ − (Ct_TARGET_ − Ct_GAPDH_)_calibrator_]. The primers were synthesized by BGI and the sequences are listed in Table 1. For each gene, the RQ value of control was designated 1.00.

**Table 1:** Primers for RT-PCR

Gene	Primer sequences (5′-3′)
*GAPDH*	Forward GGAGTCCACTGGCGTCTTCA Reverse ATTGCTGATGATCTTGAGGCTGTTG
*IL-34*	Forward CAGTACAGGAGCCGACTTCAGT Reverse ACATTCAGCAGCAGCGTCTC
*DDIT4*	Forward CGGAGGAAGACACGGCTTACC Reverse GCTTACCAACTGGCTAGGCATCA

RT-PCR = reverse transcription-polymerase chain reaction, IL-34 = Interleukin 34, DDIT4 = DNA damage-inducible transcript 4, GAPDH = glyceraldehyde-3-phosphate dehydrogenase.

### 4.7 Wright staining

Cells were spun onto slides. Wright staining was performed following the standard protocols.^36^ The morphology of cells was detected under a light microscope (AXIO Observer A1, Germany).

### 4.8 Cell proliferation assays

For the cell counting assay, 5 × 10^4^ cells were seeded per well in a 24-well plate. The cell number of each well was counted every day.

For the MTS assay, 1 × 10^4^ cells were cultured per well in a 96-well plate. At each time point, 20 μl MTS was added and cells were incubated for another 2 hours. The absorbance at 490 nm was measured by a microplate reader. Cell proliferation at a time point was assessed as the fold change in absorbance at that time point versus time point 0 hour.^2^

### 4.9 Cell cycle analysis

PI staining was used for the cell cycle analysis.^37^ Briefly, cells were collected, resuspended in phosphate-buffered saline (PBS), and fixed in 70% ethanol at 4°C for 24 hours. Then, cells were washed with PBS and RNA was digested with RNase for 15 minutes at room temperature. Finally, PI was added at the final concentration of 10 μg/ml before flow cytometry analysis.

### 4.10 Cell apoptosis analysis

The cell apoptosis analysis was described previously.^2^ Briefly, cells were collected and washed twice with binding buffer. Then, cells were resuspended in binding buffer and incubated with APC-Annexin V antibody for 15 minutes. Finally, PI was added before flow cytometry analysis.

### 4.11 Colony-forming assay

Colony-forming assays were undergone following the manufacturer's instructions.^6^ Briefly, cells were resuspended in H4434 complete medium, seeded into 24-well plates at a density of 500 cells/500 μl/well, and cultured for 7 (Molm13-IL-34) or 10 (THP1-IL-34) days. Colonies were counted under a microscope and photographed by a high-content analysis system.

### 4.12 Flow cytometry analysis and cell sorting

Flow cytometry analysis was performed following the standard protocols.^38^ Briefly, cells were harvested and stained with fluorophore-conjugated antibodies (CD11b, CD14, and CD71) for 30 minutes in dark. Subsequently, cells were washed and resuspended in PBS. Canto II and Aria III (BD Biosciences) were used for fluorescence-activated cell sortin (FACS) analysis and cell sorting, respectively. The FACS data were analyzed using the FlowJo software.

### 4.13 Statistical analysis

All experiments were repeated at least 3 times. The results were expressed as mean ± SD. The analysis was done using GraphPad Prism 8.0 software. Comparison between two groups was analyzed by unpaired Student *t* test. Statistical significance was accepted when the *P* value was <0.05.

## FUNDING

This work was supported by grants 81770183 and 81970155 from the National Natural Science Foundation of China (NSFC); programs 2016-I2M-2-006 and 2017-I2M-1-015 from the CAMS Innovation Fund for Medical Sciences (CIFMS); State Key Laboratory of Experimental Hematology Research Grant (Z20-06); G.Z. is a recipient of the New Century Excellent Talents in University (NCET-08-0329).
